# Acid-sensing ion channel blocker diminazene facilitates proton-induced excitation of afferent nerves in a similar manner that Na^+^/H^+^ exchanger blockers do

**DOI:** 10.3389/fncel.2023.1131661

**Published:** 2023-07-12

**Authors:** Yurii Tkachenko, Volodymyr Khmyz, Andrii Buta, Dmytro Isaev, Oleksandr Maximyuk, Oleg Krishtal

**Affiliations:** Bogomoletz Institute of Physiology, National Academy of Sciences of Ukraine, Kyiv, Ukraine

**Keywords:** skin-nerve preparation, primary afferent nociceptors, acidic pH, diminazene, zoniporide, 5-(N-ethyl-N-isopropyl)amiloride (EIPA), Na^+^/H^+^ exchangers (NHEs)

## Abstract

Tissue acidification causes sustained activation of primary nociceptors, which causes pain. In mammals, acid-sensing ion channels (ASICs) are the primary acid sensors; however, Na^+^/H^+^ exchangers (NHEs) and TRPV1 receptors also contribute to tissue acidification sensing. ASICs, NHEs, and TRPV1 receptors are found to be expressed in nociceptive nerve fibers. ASIC inhibitors reduce peripheral acid-induced hyperalgesia and suppress inflammatory pain. Also, it was shown that pharmacological inhibition of NHE1 promotes nociceptive behavior in acute pain models, whereas inhibition of TRPV1 receptors gives relief. The murine skin-nerve preparation was used in this study to assess the activation of native polymodal nociceptors by mild acidification (pH 6.1). We have found that diminazene, a well-known antagonist of ASICs did not suppress pH-induced activation of CMH-fibers at concentrations as high as 25 μM. Moreover, at 100 μM, it induces the potentiation of the fibers’ response to acidic pH. At the same time, this concentration virtually completely inhibited ASIC currents in mouse dorsal root ganglia (DRG) neurons (IC_50_ = 17.0 ± 4.5 μM). Non-selective ASICs and NHEs inhibitor EIPA (5-(N-ethyl-N-isopropyl)amiloride) at 10 μM, as well as selective NHE1 inhibitor zoniporide at 0.5 μM induced qualitatively the same effects as 100 μM of diminazene. Our results indicate that excitation of afferent nerve terminals induced by mild acidification occurs mainly due to the NHE1, rather than acid-sensing ion channels. At high concentrations, diminazene acts as a weak blocker of the NHE. It lacks chemical similarity with amiloride, EIPA, and zoniporide, so it may represent a novel structural motif for the development of NHE antagonists. However, the effect of diminazene on the acid-induced excitation of primary nociceptors remains enigmatic and requires additional investigations.

## Introduction

Virtually all proteins, including enzymes and ion channels, depend on pH to maintain their function (Whitten et al., 2005). Proteins gain proton sensitivity by changing the charge of amino acids by protonation or deprotonation. Only a few amino acids, such as histidine, aspartic acid, arginine, lysine, and glutamic acid, are protonated at pH values ranging from 5 to 7.4. Histidine has pKa ≈ 6–7 making it the most prominent candidate for sensing slight changes in pH. Histidine is thought to be uncharged at neutral pH and doubly protonated and positively charged at pH 6 and lower, while the effective pKa of a single histidine varies on its local environment. Tight control of both extra- and intracellular pH is essential for maintaining cellular biochemical reactions and tissue homeostasis (Casey et al., 2010). Despite the efficient pH control mechanisms, various pathological conditions, such as inflammation, injury, or solid tumors, are associated with the development of local acidosis. In the normal state, pH values in tissue ranged from 7.35 to 7.45. However, inflammation could reduce the pH in the injury place down to 6–6.5 units (Rajamaki et al., 2013). The value of extracellular pH decreases to 6.69–6.89 in fractures (Berkmann et al., 2020), intra-abdominal infections cause a drop in pH to values less than 7.1 (Simmen et al., 1994), and in various tumors, the pH ranges from 6.4 to 7.0 (Engin et al., 1995). The metabolic decrease in pH in pathological conditions is thought to be due to the accumulation of lactic acid and carbon dioxide in the affected tissues.

Intracellular pH (pHi) is a fundamental parameter of cell function that requires tight homeostasis (Casey et al., 2010). Mammalian cells constantly have to eliminate internal acidification. This task is mainly performed by Na^+^/H^+^ antiporters, which exchange extracellular Na^+^ for intracellular H^+^, which activates when cells become acidic. NHEs are widespread in neuronal and non-neuronal cells. Of the 9 NHE isoforms identified, NHE1 is the most ubiquitous and expressed in nerve cells (Slepkov et al., 2007; Torres-Lopez et al., 2013). NHE inhibitors concentration-dependently produce intracellular acidosis and can even cause cell death (Schneider et al., 2004). By using the *in vitro* electrophysiological skin-nerve preparation recording method, it was shown that intracellular proton release by photolysis from NPE-caged proton compounds caused the same irritation and transient heat sensitization of sensory endings in skin sensory neurons as extracellular acidification by phosphate buffered solution (pH 5.4) and CO_2_-gassed solution (pH 6.1) (Guenther et al., 1999). While, the membrane-permeable proton buffer SNARF-AM, which was used to prevent changes in intracellular pH, prevented pH-induced heat sensitization. Studies using fluorescent indicators have shown that depolarization with high K^+^, as well as extracellular acidification of sensory neurons, causes a decrease in intracellular pH (Werth and Thayer, 1994; Zeilhofer et al., 1997). Due to the widespread expression of NHE1 in nerve cells, including nociceptors, it is probable that NHE1 could affect the excitability of neurons and the transmission of pain by altering the pH levels within the cells. However, the experimental data on the effect of NHE blockers on the activity of nerve cells obtained using various models are somewhat contradictory. Administration of amiloride derivatives as non-selective NHE inhibitors, and zoniporide, a selective NHE1 inhibitor, significantly increased formalin-induced startle in a dose-dependent manner in rats (Castaneda-Corral et al., 2011). But, experiments performed on dorsal root ganglia (DRG) cells have shown that NHE1 inhibitor zoniporide reduces the amplitude of the compound action potential (Liu and Somps, 2008).

Pain stimuli (chemical, mechanical, and thermal) are first detected by the nerve endings of specialized peripheral primary sensory neurons called nociceptors. These nerve fibers arise from small-diameter pseudounipolar neurons located in the DRG. Endings of primary sensory neurons detect local changes in pH and respond to these changes with a depolarization response, which is thought to be the initial trigger for pain sensation. The tissue acidification causes the excitation of cutaneous nociceptors without signs of desensitization or adaptation (Steen et al., 1995). The subpopulation of polymodal nociceptive mechanoheat-sensitive C-fibers (CMH-fibers) possesses the highest sensitivity to a decrease in pH. Its threshold levels were found within the range from pH 6.9 to 6.1, and the mean maximum discharge level was at pH 5.2 (Steen et al., 1992). To detect changes in pH, nociceptors are equipped with an assortment of different acid sensors, some of which can detect mild changes in pH, such as the acid-sensing ion channels, proton-sensing G protein-coupled receptors, and several two-pore potassium channels, whereas others, such as the transient receptor potential vanilloid 1 ion channel (TRPV1), require larger shifts in pH (Pattison et al., 2019). In many species, nociceptors are polymodal, i.e., they respond to multiple noxious stimuli (e.g., heat, pressure, and chemicals such as acid), owing to the expression of different receptors (Boscardin et al., 2016).

Acid-sensing ion channels (ASICs) and TRPV1 receptors have been proposed as the main acid sensors (Ugawa et al., 2002) in nociceptors. ASIC1a, ASIC1b, ASIC3, and TRPV1 receptors are mainly expressed in small-diameter DRG neurons with a high level of co-expression. ASIC1a transcripts were found in 20–25% of DRG neurons; ASIC1b and ASIC3 transcripts are expressed in approximately 10 and 30–35% of DRG neurons, respectively (Ugawa et al., 2005). Half-maximal activation (pH_50_) for ASIC1a occurs at pH 6.2–6.6; for ASIC1b, pH_50_ is 5.9–6.3; and pH_50_ for ASIC3 is 6.4–6.7 (Boscardin et al., 2016). Severe extracellular acidosis (acidification to pH ≤ 6.4) activates the whole repertoire of ASIC subtypes. TRPV1 is expressed in 35–40% of DRG neurons (Ugawa et al., 2005). TRPV1 is a polymodal ion channel activated by capsaicin and a number of other stimuli including noxious heat (>42°C) and protons (Caterina et al., 1997; Mickle et al., 2015). TRPV1-mediated sustained currents due to acidification have a pH_50_ of ∼5.4. Thus, TRPV1 and ASICs ion channels expressed in small-diameter DRG neurons are very important extracellular acid sensors, contributing to acid-induced nociception within the physiological pH range (below pH 7.3 for ASICs and below pH 6.0 for TRPV1) (Blanchard and Kellenberger, 2011).

The high sensitivity of ASICs to acidosis and their distribution in primary sensory neurons point to a significant role of these channels in acid-induced nociception (Kellenberger and Schild, 2002; Krishtal, 2003). Local administration of ASIC1 and ASIC3 channel activators induces pain behavior (Yu et al., 2010; Bohlen et al., 2011), while the administration of ASIC channel inhibitors has an analgesic effect (Jones et al., 2004; Diochot et al., 2012). These data indicate that ASICs are promising targets for antinociceptive drugs. However, a well-known blocker of ASICs amiloride demonstrates some controversial effects on different experimental models (Steen et al., 1999; Ugawa et al., 2002). In this study, we examined the effect of the non-amiloride ASICs receptor antagonist, diminazene, on acid-evoked responses of mouse cutaneous nociceptors *ex vivo*.

## Materials and methods

### Animals

Adult (6–7 weeks old) BALB/c mice weighing 22–30 g were used in the *ex vivo* skin-nerve preparation experiments. For patch-clamp studies, mice aged 8–12 days were selected. To avoid potential sex-specific effects (Riley et al., 1998; Ross et al., 2018) only male subjects were used in all the experiments. Animals were bred in the vivarium of the Bogomoletz Institute of Physiology, where they were housed on a 12-h light-dark cycle and given food and water *ad libitum*. All experiments were performed in accordance with the guidelines of the Bogomoletz Institute Animal Care and Use Committee. A total of 34 mice were used in this study.

### Skin-nerve preparation

Murine skin-nerve preparation has been used for electrophysiology recording from single primary afferents, as described earlier (Tkachenko et al., 2023). Under deep anesthesia by urethane (2 g per kg, i.p.), the saphenous nerve and its innervating territory on the hairy hind paw skin were subcutaneously dissected and excised. After dissection, the animals were euthanized with an intraperitoneal injection of a lethal dose of urethane. Next, the skin was gently stretched and pinned corium side up in the organ bath for pharmacological application to the receptive fields of single sensory units. The end of the nerve was gently threaded through a hole into a separate recording chamber for fiber teasing and single-unit recording. The tissue and recording chambers were separately superfused with a modified Krebs–Henseleit solution composed of (in mM): NaCl 118, KCl 5.4, NaH_2_PO_4_ 1.0, MgSO_4_ 1.2, CaCl_2_ 1.9, NaHCO_3_ 25.0, and dextrose 11.1, and gassed with 95% O_2_–5% CO_2_, pH 7.4, at a flow rate of 8 ml × min^–1^ and 3 ml × min^–1^, respectively. The temperature in the organ bath and recording chamber was maintained at 32 ± 0.1°C.

### Single fiber activity recordings

**FIGURE 1:**
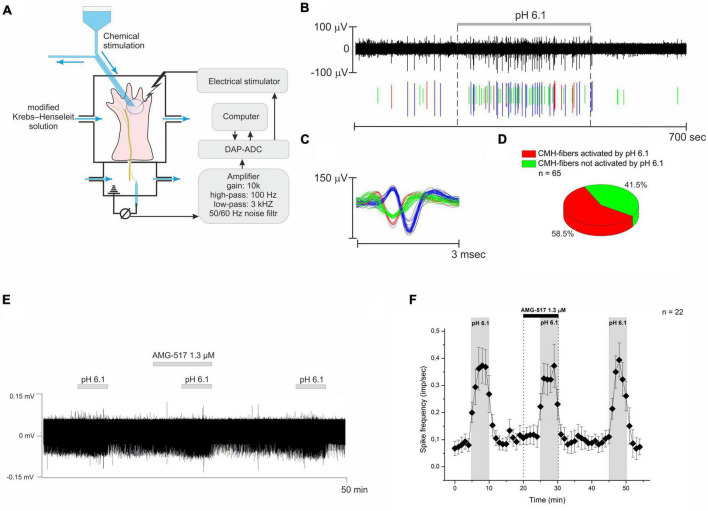
**(A)** The simplified experiment diagram shows the process of identifying the receptive fields of nerve fibers using electrical, mechanical, and temperature stimulation. Next, the skin area with the receptive field was isolated using a small glass ring for chemical stimulation and drug testing. **(B)** Example recording from three nervous CMH-fibers upon stimulation of their receptive field using a modified Krebs–Henseleit solution with pH 6.1. **(C)** Action potentials from the same nerve filament are displayed as their superimposed waveforms. **(D)** Summary data for activation of CMH-fibers by pH 6.1. **(E)** Representative recording of CMH-fiber activity under the TRPV1 receptor antagonist AMG-517. AMG-517 was applied at a concentration of 1.3 μM before and during the activation of nerve fibers by pH 6.1 at 40°C. **(F)** Descriptive statistics: CMH-fiber activity (imp/s) was averaged at 60-s intervals (mean ± SEM). AMG-517 did not alter the activity of CMH-fibers by itself, nor did it cause any changes in low pH-induced discharge.

The recordings of single fiber activity were performed using a suction glass microelectrode. The microelectrodes were pulled using a Flaming-Brown micropipette puller (Model P-97, Sutter Instrument CO., USA). The recording signal was amplified (Model 3000, A-M Systems, Inc., Carlsborg, WA, USA) and filtered (low cut-off, 0.3 kHz; high cut-off, 1 kHz). Nerve fiber activities were recorded to a PC via a Digidata 1200 analog-to-digital converter (Axon Instruments, USA). Single units were characterized as follows: conduction latency in milliseconds was determined by electrical stimulation inside the receptive field with a fine steel electrode using an A-M Systems analog stimulus isolator model 2200, and the conduction distance was assessed to calculate conduction velocity. A cutoff of 1.0 m/s was used to distinguish between myelinated and unmyelinated fibers. Extracellular recordings were obtained only from single fibers that could be easily discriminated according to amplitude and shape (Figure 1A). We limited our investigation to the CMH subpopulation of nerve fibers since this group of mechano-heat sensitive “polymodal” C-units is significantly more responsive to acidic pH (Steen et al., 1992). Other types of fibers, including the A-fiber population with higher conduction velocities, were not investigated. The CMH nerve fibers that did not respond to chemical stimulation were not taken into account. The recorded signal was stored on a computer using WinEDR v 3.3.1 software.

### Chemical stimulation of the nerve endings

To boost the rate of solution exchange, the receptive field was isolated from its surroundings using a tiny glass ring (inner diameter: 6 mm; volume: 0.3 ml). The ring was separately perfused at 4 ml/min, providing a rapid exchange of the fluid. The nerve fiber’s receptive field was superfused with the same modified Krebs-Henseleit solution, continuously gassed with 100% CO_2_, triggering a pH drop to 6.1 (Steen et al., 1992). The experimental series consisted of three subsequent acidic stimuli of a 5-min duration. After a 5-min pre-application, the tested reagents were applied together with the second acid stimulus. Each chemical stimulation was followed by a washout period lasting more than 10 min. The temperature of the buffer solutions in the glass ring was maintained at 32 ± 0.1°C unless otherwise indicated.

### Primary culture of mouse DRG neurons

Dorsal root ganglia neurons were isolated using a standard procedure (Maximyuk et al., 2015). In brief, after animal decapitation, ganglia were rapidly removed and placed in an Eagle’s minimal essential medium (MEM) solution containing 4 mg/mL trypsin and 2 mg/mL collagenase for 25 min. The solution was held at 35°C during the enzymatic treatment and constantly saturated with a gas mixture of 95% oxygen and 5% carbon dioxide to maintain a pH of 7.4. The ganglia were then rinsed out and dissociated in a MEM solution containing 7.4 pH HEPES-NaOH. Finally, the isolated neurons were suspended in a mixture of 90% Dulbecco’s modified Eagle’s medium (DMEM), 0.3% penicillin, 10 μg/ml insulin, and 10% fetal calf serum and maintained at 37°C for 5–48 h before being used in electrophysiological experiments.

### Patch-clamp experiments

The whole-cell patch clamp recordings were done using an EPC8 amplifier and LIH 1600 acquisition system (HEKA, Germany). The recordings were taken at a holding potential of −60 mV. The current traces were sampled at 10–20 kHz and filtered online at 7 kHz. The patch electrodes, having a resistance of 2–3 MΩ, were filled with a solution containing 120 mM CsF and 20 mM Tris-Cl, pH 7.3. The extracellular solution had the following composition: 130 mM NaCl, 5 mM KCl, 2 mM MgCl_2_, 2 mM CaCl_2_, and 20 mM HEPES/NaOH, pH 7.4 (adjusted with NaOH). A rapid pH decrease from 7.4 to 6.0 caused a transient transmembrane ionic current, which is mediated by activation of ASICs, in around 30% of the DRG neurons examined. PatchMaster v.2 × 69 software (HEKA, Germany) was used to record and store the experimental data. A fully automated “jumping table” setup (PharmaRobot in Kyiv, Ukraine) was used for solution exchange, allowing for approximately 90% solution exchange in about 10 ms. The experiments were made at room temperature (20°C).

### Data analysis

Single-fiber recordings were analyzed offline with a template-matching function of Spike 2 software (CED, Cambridge, UK). The PatchMaster v.2 × 69 (HEKA, Germany) software was used for the initial analysis of patch-clamp data. Origin Pro 8.5 software (Origin Lab. Corp., Northampton, MA, USA) was used for conducting all statistical tests. All experimental data are presented as mean ± SEM. A paired Student’s *t*-test was used to determine the significance of the differences in nerve fiber activity and for the comparison of current amplitudes in patch-clamp experiments. The level of significance was chosen at 0.05 unless otherwise indicated.

### Chemicals

All chemicals were purchased from Sigma-Aldrich Chemie GmbH (Taufkirchen, Germany).

## Results

Single-unit recordings were made from a total of 87 CMH-fibers of polymodal nerve afferents from 32 animals. All tested CMH-fibers demonstrate the spontaneous basal activity with a corresponding instantaneous frequency of 0.034 ± 0.0083 imp/s at 32°C.

In response to the stimulation of the receptive field by pH 6.1, an increase in spontaneous activity to 0.449 ± 0.099 imp/s (*n* = 52, *p* = 0.000073, Figures 1B, C) was observed in 58.5% (38/65, Figure 1D) of nerve fibers. Further decreasing pH promoted an increase in CMH-fiber activity as well as the percent of responding fibers (data not shown). However, TRPV1 receptors are also sensitive to extracellular pH and may induce the firing of nerve fibers in response to low pH (Tominaga et al., 1998; Blanchard and Kellenberger, 2011). We have checked the effect of a selective antagonist of TRPV1 channels, AMG-517, on the response of CMH-fibers to pH 6.1 at 40°C. The tested CMH-fibers (*n* = 22) demonstrated spontaneous basal activity with a corresponding instantaneous frequency of 0.08 ± 0.025 imp/s in control conditions at the beginning of the experiment. AMG-517 1.3 μM by itself did not affect the basal activity of nerve fibers during pre-application (*n* = 22, *p* = 0.096, Figures 3E, F). Stimulation by pH 6.1 increased spike frequency to 0.51 ± 0.084 imp/s (*n* = 22, *p* = 0.000009), while AMG-517 at a concentration of 1.3 μM did not produce any feasible effect on this increase (0.483 ± 0.074; *n* = 22; *p* = 0.298).

### Diminazene does not inhibit the acid-induced activity of CMH-fibers at 10 and 25 μM

**FIGURE 2:**
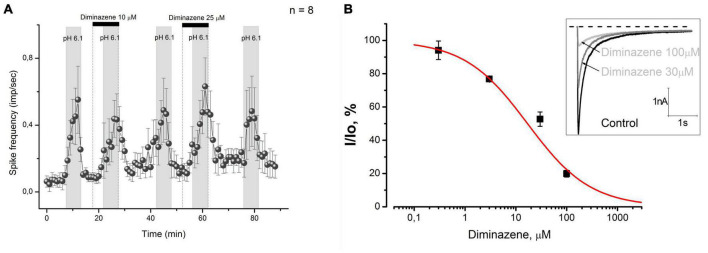
**(A)** Diminazene at concentrations of 10 and 25 μM did not excite CMH-fibers by itself and did not cause changes in low pH-induced discharge. **(B)** Diminazene inhibits native ASIC currents in primary cultured mouse DRG neurons at micromolar concentrations. Currents were elicited by a rapid pH drop from 7.4 to 6.0 at a holding voltage of –60 mV. The dose-response fit of the data gives IC_50_ = 16.9 ± 4.4 μM and *k* = 0.7 ± 0.1.

Acid-sensing ion channels are shown to be the most sensitive effectors to protons (Ugawa et al., 2002; Blanchard and Kellenberger, 2011) in sensory nerve endings, while diminazene has been proven to be an efficient blocker of them [pIC50 ∼ 6.5, see Chen et al. (2010)]. Therefore, the diminazene should effectively antagonize the response of the CMH-fibers to acidic pH. We examined the effect of diminazene in concentrations of 10 and 25 μM on the response of CMH-fibers to pH 6.1. The tested CMH-fibers (*n* = 8) demonstrated spontaneous basal activity with a corresponding instantaneous frequency of 0.063 ± 0.031 imp/s in control conditions at the beginning of the experiment. Diminazene by itself did not affect the basal activity of nerve fibers during pre-application (*n* = 8, *p* = 0.291, and *p* = 0.14, correspondingly, Figure 2A). Stimulation by an acidic pH increased spike frequency to 0.683 ± 0.17 imp/s (*n* = 8, *p* = 0.00416, Figure 2A). Coadministration of acidic solution with diminazene at concentrations as high as 10 and 25 μM did not produce any feasible effect on this increase (0,533 ± 0.117; *n* = 8; *p* = 0.157, and 0.679 ± 0.174 imp/s *n* = 8; *p* = 0.972, correspondingly).

Since diminazene at concentrations as high as 10 and 25 μM did not affect the acid-induced response of skin nociceptors, we used the patch-clamp technique to evaluate its activity on native acid-sensing ion channels expressed in mouse DRG neurons, because we could not find any data on its activity on these channels. We have found that diminazene causes a dose-dependent inhibition (Figure 2B) of the ASIC currents in mouse DRG neurons with a corresponding IC_50_ = 16.9 ± 4.4 μM, which is substantially higher than previously reported for various ASIC subunits (Chen et al., 2010).

So, diminazene effectively decreased native ASICs expressed in the cell bodies of mouse DRG neurons at high micromolar concentrations. The ASIC currents in these neurons were almost completely suppressed by 100 μM diminazene.

### Diminazene facilitates acid-induced activity of CMH-fibers at 100 μM

**FIGURE 3:**
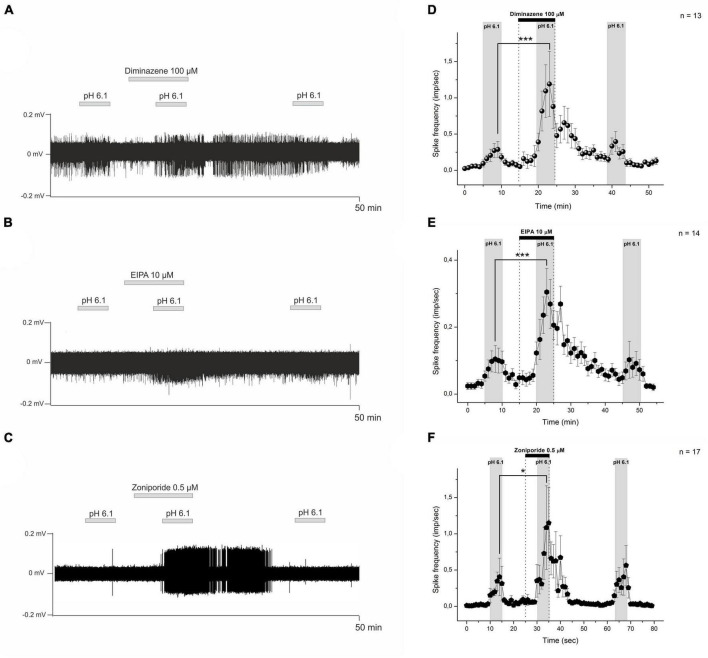
Sample recordings from nerve fibers show the influence of diminazene 100 μM **(A)**, EIPA 10 μM **(B)**, and zoniporide 0.5 μM **(C)** on the low pH-induced nociceptor discharge. The test drugs were applied with an acidic solution (pH 6.1) to the receptive field of nerve fibers. Descriptive statistics **(D–F)**. The graphs show averaged CMH-fiber activity (imp/s) at 60-s intervals (mean ± SEM). **(D)** Diminazene 100 μM did not excite the nociceptors at pH 7.4, while low pH-induced discharge increased under diminazene. Polymodal C-fibers also show an increase in low pH-induced discharge under EIPA 10 μM **(E)** and zoniporide 0.5 μM **(F)**. *p < 0.05, ***p < 0.001.

Our findings on diminazene activity on native ASICs expressed in mouse DRG neurons prompted us to increase its concentration in the skin-nerve preparation experiments. In this series of experiments, the tested CMH-fibers (*n* = 13) demonstrated spontaneous baseline activity with a corresponding instantaneous frequency of 0.046 ± 0.02 imp/s in control conditions. Stimulation by the acidic pH statistically significantly increased the average spike frequency to 0.398 ± 0.106 imp/s (*n* = 13, *p* = 0.00265). Diminazene at a concentration of 100 μM did not excite nociceptors by itself while applied alone (*n* = 13, *p* = 0.281, Figure 3A), while its coadministration with a low-pH solution increased the low pH-induced activity to 1.806 ± 0.416 imp/s (*n* = 13, *p* = 0.000337, Figures 3A, D). Our data indicate that diminazene at a concentration of 100 μM increased the responsiveness of CMH-fibers to pH 6.1 by 453.8%.

The above, together with the patch-clamp data, pointed us to the hypothesis that the enhancement of acid-induced nerve fibers responses by diminazene involves excitation mechanisms not associated with the activation of ASICs located on nerve endings. It was reported that the ASIC and NHE blocker amiloride increases the pH response of rat skin nociceptors in a dose-dependent manner (Steen et al., 1999). One would suggest that in our experiments, diminazene acts similarly to amiloride, enhancing the response of mouse skin nociceptors to low pH. To verify this hypothesis we tested the effect of well-known NHE1 antagonists on acid-induced responses.

### Facilitation of acid-induced activity of CMH-fibers by NHE blockers EIPA and zoniporide

Recently, it was shown that EIPA acts as a non-specific ASIC and NHEs inhibitor (Leng et al., 2016). In these experiments, 14 tested CMH-fibers demonstrated spontaneous activity with a corresponding instantaneous frequency of 0.027 ± 0.01 imp/s in control conditions. Similarly to diminazene, EIPA at 10 μM did affect nociceptors by itself during pre-application (*p* = 0.14, Figures 3B, E). Stimulation by the acidic pH statistically significantly increased the spike frequency to 0.11 ± 0.028 imp/s (*n* = 14, *p* = 0.023). Application of EIPA at 10 μM together with a low-pH solution increased the acid-induced activity to 0.414 ± 0.064 imp/s (*n* = 14; *p* = 0.00003; Figures 3B, E). Four nerve fibers were totally insensitive to both low pH and low pH supplemented with 10 μM (22%). In summary, EIPA at a concentration of 10 μM causes increased sensitivity to low pH of CMH-fibers by 376.4%.

Unlike EIPA, zoniporide is a selective and potent sodium-hydrogen exchanger isoform 1 (NHE1) inhibitor. All tested CMH-fibers demonstrate basal spontaneous activity with corresponding instant frequency of 0.017 ± 0.0057 imp/s (*n* = 17) in control conditions. Stimulation by the acidic pH statistically significantly, increased spike frequency to 0.661 ± 0.272 imp/s (*n* = 17, *p* = 0.029). Zoniporide at 0.5 μM did not affect the spontaneous activity of primary nerve afferents during pre-application by itself (*n* = 17, *p* = 0.154). Whereas, an application of 0.5 μM zoniporide together with the low pH solution increased the acid-induced activity to 1.678 ± 0.58 imp/s (*n* = 17, *p* = 0.0118, Figures 3C, F). So, zoniporide at a concentration of 0.5 μM causes an increase in sensitivity to the low pH of CMH-fibers of 253.8%. Thus, the combination of zoniporide 0.5 μM with low pH induces significantly higher excitation of cutaneous afferent nerve endings than EIPA, but not diminazene.

## Discussion

The decrease in pH to the threshold level of 6.9 to 6.1 causes the excitation of cutaneous nociceptors without signs of desensitization or adaptation (Steen et al., 1992). In our experiments, lowering the pH of the superfusion solution from 7.4 to 6.1 induced an approximately 13-fold increase in the spontaneous activity of CMH-fibers in 58.5% (Figures 1B, D) cases. While ASICs are described as the most sensitive sensors of protons in nociceptors (Lee and Chen, 2018), their activation is suspected to be the main mechanism for sensing the elevated concentration of protons by nociceptors. However, TRPV1 receptors are also sensitive to extracellular hydrogen ions and may cause the firing of nerve fibers in response to low pH [below 6.0 (Blanchard and Kellenberger, 2011)], especially at elevated temperatures (Tominaga et al., 1998; Tkachenko et al., 2023). To exclude this hypothesis, we used AMG-517, a selective antagonist of TRPV1 channels. At the concentration of 1.3 μM, AMG-517 did not affect the described acid sensitivity of the skin nerve fibers (Figure 1F), indicating that TRPV1 channels are not involved in the observed excitation of nerve fibers.

Diminazene was previously known as an anti-protozoal drug in veterinary medicine, but it is now one of the most potent commercially available low-molecular-weight inhibitors of ASIC. It is a non-amiloride-derived ASIC that inhibits ASIC currents at submicromolar concentrations via an open channel and subtype-dependent mechanism and, unlike amiloride, does not have an effect on ENaC (Chen et al., 2010; Lee et al., 2018). The highest potency of diminazene was found to be for ASIC1b and ASIC3, both of which are highly expressed in peripheral sensory neurons and implicated in peripheral nociception. Consistent with this, it was found that diminazene induces peripheral antihyperalgesia in Freund’s complete adjuvant rat model of unilateral inflammatory pain, but substantial inter-individual heterogeneity in the antihyperalgesic efficacy of ASIC inhibition was also revealed: some animals (more than 50%) were totally insensitive to the blockade of ASICs (Lee et al., 2018). We found that the blockade of ASICs by diminazene in concentrations as high as 10 and 25 μM did not affect the low pH-induced responses in sensory nerve fibers (Figure 2A). The blocking effect of diminazene was described in ASICs heterologously expressed in CHO cells, *X. laevis* oocytes, and native rat channels (Chen et al., 2010; Lee et al., 2018), but not for mouse channels. Consequently, we decided to test the sensitivity of native mouse ASICs expressed in DRG neurons to diminazene. In our experiments, mouse ASICs were found to be at least 20-fold less sensitive to diminazene: IC_50_ ∼ 320 and 864 nM for rASIC3 and rASIC2a correspondingly (Lee et al., 2018) vs. IC_50_ = 16.9 ± 4.4 μÌ (Figure 2B). Respectively, we have decided to increase its concentration in sensory nerve excitation experiments but found an unpredictable result: the low pH-induced activity of nerve fibers was significantly increased under 100 μM of diminazene (0.398 ± 0.106 vs. 1.806 ± 0.416 imp/s; *n* = 13, *p* = 0.000337, Figures 3A, D). So, diminazene at a concentration of 100 μM increased the responsiveness of CMH-fibers to pH 6.1 by 453.8%. The described action of diminazene was qualitatively the same as that previously described for amiloride (Steen et al., 1999).

Amiloride and its analogs, such as EIPA, are non-specific blockers of ASICs and Na^+^/H^+^ exchangers (Kleyman and Cragoe, 1988; Maidorn et al., 1993; Leng et al., 2016). Amiloride is well-known blocker of ASICs (Leng et al., 2016). On the other hand, amiloride causes a dose-dependent increase and prolongation of low pH-induced responses in rat skin-nerve preparation (Steen et al., 1999). Inhibition of the Na^+^/H^+^ antiporter by replacing external sodium with sucrose induces the same effect on the onset of the response as its blocking by amiloride. The authors conclude that in both cases, blockade of the Na^+^/H^+^ antiporter increased intracellular acidosis in nerve endings and, as a consequence, increased nociceptive activity (Steen et al., 1999). Peripheral and spinal administration of the non-selective NHE inhibitors amiloride, 5-(N,N-dimethyl)amiloride (DMA), EIPA, and the selective NHE1 inhibitor zoniporide significantly increased formalin-induced nociceptive behavior in a dose-dependent manner (Rocha-Gonzalez et al., 2009; Castaneda-Corral et al., 2011). This data suggests that the sodium*-*proton exchangers, especially their first isoform (NHE1), play a role in pain processing at peripheral and spinal levels in formalin-induced long-lasting nociceptive behaviors.

Our data indicate that the application of 10 μM EIPA and 0.5 μM of the selective NHE1 blocker zoniporide produces qualitatively the same effects as we described for diminazene. The instantaneous frequency of the response to low pH rose in response to the application of 10 μM EIPA from 0.11 ± 0.028 to 0.414 ± 0.064 imp/s (*n* = 14; *p* = 0.00003; Figures 3B, E). Thus, EIPA at a concentration of 10 μM causes increased sensitivity to low pH of CMH-fibers by 376.4%. EIPA is a derivative of amiloride and is not the selective antagonist of Na^+^/H^+^ antiporter. It is known that the amiloride derivative EIPA also acts as a weak blocker of ASIC receptors (Leng et al., 2016).

Therefore we used a selective blocker of a widely distributed first isoform of Na^+^/H^+^ antiporter (NHE1) zoniporide. Zoniporide at a concentration of 1 μM is sufficient for the effective inhibition of NHE1 (Kleyman and Cragoe, 1988). Moreover, at this concentration, it did not induce any changes in the activity of ASIC channels endogenously expressed in DRG neurons of mice and rats as well as on hASIC1a in HEK293 cells (Supplementary Figure 1). Here we show that in the concentration of 0.5 μM zoniporide facilitates the low pH-induced response from 0.661 ± 0.272 to 1.678 ± 0.58 imp/s (*n* = 17; *p* = 0.0118; Figure 3F) providing the increase in sensitivity of CMH-fibers to low pH by 253.8%. Taken together these data suggest that diminazene may act as a weak blocker of Na^+^/H^+^ antiporter.

Our data support previous studies and demonstrate that blockers of sodium/hydrogen exchangers (NHEs) increase the nociceptive response of nerve fibers. NHEs are expressed in various tissues of the body in many species. According to a widely accepted model, NHEs located on the gill epithelium of fish play a crucial role in sodium uptake. It was shown that amiloride and diminazene dose-dependently reduce Na^+^ uptake in adult rainbow trout at micromolar concentrations (Dymowska et al., 2014). In our study, diminazene enhanced the response to low pH stimulation in mouse nerve fibers. This suggests that diminazene may also influence proton transport out of the nerve endings. It is worth noting that certain drugs can bind to multiple molecular targets or receptors, resulting in a broad range of side effects (Frantz, 2005). So we can speculate that diminazene acts as a weak blocker of the NHE. It lacks chemical similarity with amiloride, EIPA, and zoniporide, so it may represent a novel structural motif for the development of HNE antagonists. But we could not exclude that the effect of diminazene in high concentrations is totally unspecific between different effectors due to the presence of two amidine functional groups. These highly charged groups can provide non-specific effects, and novel designs of ASICs antagonists should consider this issue. In summary, the effect of diminazene on the acid-induced excitation of primary nociceptors remains enigmatic and requires additional investigations.

## Data availability statement

The original contributions presented in the study are included in the article/Supplementary material, further inquiries can be directed to the corresponding author.

## Ethics statement

The animal study was reviewed and approved by the Bogomoletz Institute Animal Care and Use Committee.

## Author contributions

YT conducted experimental research using the method of skin-nerve preparation, experimental data processing, and analysis of experimental data. VK conducted experimental research using the method of patch-clamp, analysis, and interpretation of experimental data. AB conducted experimental research using the method of patch-clamp. DI substantial contribution to the conception and design of experiments. OM conception of research, wrote the manuscript, data analysis, and interpretation. OK project management and critical review of the relevance of experimental research. All authors contributed to the article and approved the submitted version.
