# Supplementary material for: Acid-sensing ion channel blocker diminazene facilitates proton-induced excitation of afferent nerves in a similar manner that Na+/H+ exchanger blockers do
Source: Front Cell Neurosci. 2023 Jul 12;17:1131661. doi: 10.3389/fncel.2023.1131661 (PMC10368877; doi:10.3389/fncel.2023.1131661)
Supplement: Supplementary file 1 [file Image_1.PDF]

Supplementary Methods

Primary culture of rat DRG neurons

Male rats aged 8-12 days were used for patch-clamp experiments. Animals were bred in the vivarium of the Bogomoletz Institute of Physiology, where they were housed on a 12-hour light-dark cycle and given food and water *ad libitum*. All experiments were performed in accordance with the guidelines of the Bogomoletz Institute Animal Care and Use Committee. DRG neurons were isolated using a standard procedure (Maximyuk et al., 2015). In brief, after animal decapitation, ganglia were rapidly removed and placed in an Eagle's minimal essential medium (MEM) solution containing 4 mg/mL trypsin and 2 mg/mL collagenase for 25 minutes. The solution was held at 35°C during the enzymatic treatment and constantly saturated with a gas mixture of 95% oxygen and 5% carbon dioxide to maintain a pH of 7.4. The ganglia were then rinsed out and dissociated in a MEM solution containing 7.4 pH HEPES-NaOH. Finally, the isolated neurons were suspended in a mixture of 90% Dulbecco's modified Eagle's medium (DMEM), 0.3% penicillin, 10 μg/ml insulin, and 10% fetal calf serum and maintained at 37°C for 5–48 hours before being used in electrophysiological experiments.

HEK 293 cell culture

Human embryonic kidney 293 (HEK 293) cells (American Type Culture Collection, Manassas, VA, USA) expressing endogenous hASIC1a channels (Gunthorpe et al., 2001) were cultured in Dulbecco's modified Eagle medium (DMEM) supplemented with 10% fetal bovine serum (FBS) and 10 U/ml penicillin and 10 mg/ml streptomycin. Dissociated cells were either re-plated for a new passage or used for patch clamp experiments. Cells were cultured by standard procedure (Maximyuk et al., 2015) at 37 ºC under an atmosphere of 5% CO_2 and 95% air with approximately 95% humidity.

Supplementary Data

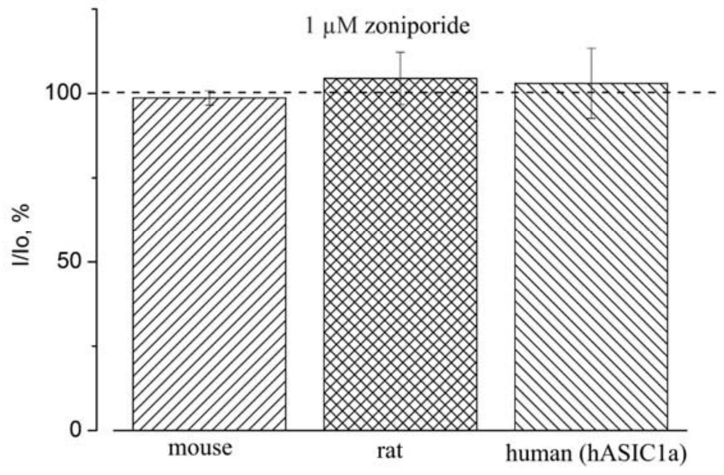

Supplementary Figure 1. Zoniporide do not affect the activity of ASICs expressed in various hosts.

In concentration as high as 1 μM zoniporide did not affected currents elicited by pH drop from 7.4 to 6.0 through ASIC channels expressed in mice DRG neurons (98.7±1.5, n=3), rat DRG neurons (104.4±5.5, n=3) and human ASIC1a channels (100.2±3.8, n=5) endogenously expressed in HEK 293 cells.
